# Comparison of methods for correcting population stratification in a genome-wide association study of rheumatoid arthritis: principal-component analysis versus multidimensional scaling

**DOI:** 10.1186/1753-6561-3-s7-s109

**Published:** 2009-12-15

**Authors:** Dai Wang, Yu Sun, Paul Stang, Jesse A Berlin, Marsha A Wilcox, Qingqin Li

**Affiliations:** 1 Department of Pharmacogenomics, Johnson & Johnson Pharmaceutical Research and Development, LLC, Raritan, New Jersey 08869 USA; 2 Department of Epidemiology, Johnson & Johnson Pharmaceutical Research and Development, LLC, Titusville, New Jersey 08560 USA

## Abstract

Population stratification (PS) represents a major challenge in genome-wide association studies. Using the Genetic Analysis Workshop 16 Problem 1 data, which include samples of rheumatoid arthritis patients and healthy controls, we compared two methods that can be used to evaluate population structure and correct PS in genome-wide association studies: the principal-component analysis method and the multidimensional-scaling method. While both methods identified similar population structures in this dataset, principal-component analysis performed slightly better than the multidimensional-scaling method in correcting for PS in genome-wide association analysis of this dataset.

## Background

In the past few years, the genome-wide association (GWA) approach has become a widely used tool for identifying genetic loci related to disease risk. Population stratification (PS) is a major challenge in GWA studies (GWAS), because of the risk of generating false positives that represent genetic differences from ancestry rather than genes associated with a disease. Among the methods developed for correcting PS in GWAS, the principal-component analysis (PCA) method [1,2] and the multidimensional-scaling (MDS) method [3,4] are also capable of detecting population structure. The PCA method identifies principal components that represent the population structure based on genetic correlations among individuals. The MDS method detects meaningful underlying dimensions that explain observed genetic distance, e.g., pairwise identity-by-state (IBS) distance, among individuals. While other methods for addressing population structure exist, we focused on these two methods in this study.

The objectives of this study were: 1) to compare the population structures identified by PCA and MDS in the rheumatoid arthritis (RA) dataset of Genetic Analysis Workshop 16 (GAW16); and 2) to evaluate the performance of these two approaches for correcting PS in GWA analyses.

## Methods

### GAW16 Problem 1 data

GAW16 Problem 1 data, provided by the North American Rheumatoid Arthritis Consortium (NARAC), contained genome-wide data on 868 RA cases and 1,194 controls. Genotype data on 545,080 single-nucleotide polymorphisms (SNPs) were available for analysis.

### Genotype data quality control

Quality control of genotype data was conducted at both the individual level and the SNP level. At the individual level, a call rate of at least 0.95 was required. Sex discrepancies were examined using the heterozygosity rate of X-chromosome. At the SNP level, a call rate of at least 0.90, a minor allele frequency of at least 0.01, and a *p*-value from the Hardy-Weinberg equilibrium test of at least 0.05/545,080 were required.

### Principal-component analysis

PCA was performed using the computer program EIGENSOFT 2.0 [1,2]. Theoretically, the leading components should reflect population structure. In this case, some of the leading components appeared to be dominated by a small set of markers all mapped to a few very small chromosome regions that showed extended linkage disequilibrium (LD). To deal with this problem, we applied a modified version of the PCA as described by Fellay et al. [5]. A first round PCA was conducted using all autosomal SNPs with minor allele frequency >0.01. SNP loadings for the leading components were compared with a normal distribution to determine whether these components depended on many SNPs across the genome or if they were dominated by relatively few SNPs all mapped to a few small chromosome regions with extended LD, as would be expected when the given component reflected population structure or a more localized LD effect, respectively. To correct for the local effects, the PCA was re-applied in a reduced SNP set. In this reduced SNP set, i) SNPs with loadings that deviated from their expected normal quantiles with a distance greater than one were excluded along all leading components; ii) remaining SNPs were pruned using the "indep-pairwise" option in PLINK 1.03 [3] such that all SNPs within a given window size of 100 had pairwise *r*^2 ^< 0.2; iii) each SNP was regressed on the previous two SNPs, and the residual entered into the PCA. SNP loadings on all components deemed significant by the Tracy-Widom statistic [6] were re-inspected to make sure that no component was dominated by a small LD region of the genome. In case there were still leading components dominated by local LD regions, the second round of PCA was repeated with adjusted parameters until no component was dominated by a small LD region. Population outliers were excluded along all significant components.

### Multidimensional scaling

MDS analysis was performed using PLINK1.03 [3]. All SNPs that passed quality control were pruned such that all SNPs within a given window size of 100 had pairwise *r*^2 ^< 0.2. Pairwise IBS distance was calculated using all autosomal SNPs that remained after pruning. Five nearest neighbors were identified for each individual based upon the pairwise IBS distance. IBS distance to each of the five nearest neighbors was then transformed into a *Z *score. Individuals with a minimum *Z *score among the five nearest neighbors less than -4 were excluded from analysis as population outliers. MDS dimensions were extracted using the "MDS-plot" option.

### Genome-wide association analyses

Three GWA analyses were performed using PLINK 1.03 [3]. These three GWA analyses were Cochran-Armitage trend test without any adjustment for PS, logistic regression with the final set of significant principal components as covariates, and logistic regression with leading MDS dimensions as covariates. The genomic inflation factor [7] was calculated for each GWA analysis.

## Results and discussion

### Genotype data quality control

All individuals had call rates >0.95 at the individual level. An examination of sex led to the exclusion of seven individuals due to incorrect or ambiguous sex information when compared with phenotype data. At the SNP level, 5,449 SNPs with call rates <0.90, 23,205 SNPs with minor allele frequencies <0.01, 1,389 SNPs with *p*-values from Hardy-Weinberg equilibrium test <0.05/545,080, and 10 SNPs on Y-chromosome were excluded from analysis. After genotype data quality control, there were 2,055 subjects and 515,741 SNPs in the analysis dataset.

### PCA, MDS, and population structure

In the first round of PCA, 59 components met the criteria for statistical significance using the Tracy-wisdom statistic. Nearly half of the SNPs that deviated from their expected normal quantiles with a distance of at least 1 (4,413 of 9,980) were in the HLA region (chr6: 25,000,000-33,500,000 bp), a region that had been reported with higher genetic heterogeneity across different populations. After removing SNPs that deviated from their expected normal quantiles with a distance of at least one and pruning the SNPs based on LD information, 81,636 autosomal SNPs were included in the second round of PCA. This analysis resulted in eight significant components using the Tracy-Widom statistic. A small number of individuals (*n *= 9) were excluded as outliers with scores on one of the significant principal components more than six standard deviations beyond the sample mean score.

In the MDS analysis, 81,652 autosomal SNPs were used to calculate the pairwise IBS distance after SNP pruning. A small number of individuals (*n *= 7) were excluded as outliers with a minimum Z score among five nearest neighbors less than -4. Among the seven outliers, five were also among the nine outliers excluded by PCA. The first eight dimensions were retained for correcting the PS in GWA analysis.

**Figure 1:**
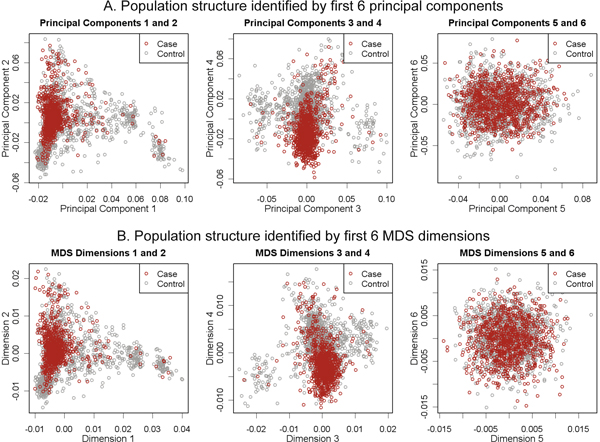
**Population structures identified by PCA and MDS**. A, The first six principal components are plotted against one another with RA status distinguished by shading; B, the first six MDS dimensions are plotted against one another with RA status distinguished by shading.

The Pearson correlation coefficients between each of the eight significant principal components and each of the eight leading MDS dimensions are summarized in Table 1. The first four principal components were strongly correlated with the first four MDS dimensions, respectively. The correlations started to drop from the fifth principal components and the fifth MDS dimensions. To illustrate the population structures identified by the two methods, the first six principal components (dimensions) were plotted against one another with RA status distinguished by shading in Figure 1. Obvious population structures were observed in the plots of the first four principal components (dimensions), but not in the plots of the fifth and sixth principal components (dimensions). In addition, the population structure identified by the first four principal components and the structure identified by the first four MDS dimensions were very similar. These results suggested that both PCA and MDS were able to detect the major population structure in this dataset with the first four principal components or the first four MDS dimensions. The population structures detected by the remaining significant principal components or leading MDS dimensions were subtle. The two methods could detect different aspects of the subtle population structure in this dataset.

**Table 1:** Correlation between first eight principal components and first eight MDS dimensions

Top eight principal components	Top eight MDS dimensions							
	
	dim1	dim2	dim3	dim4	dim5	dim6	dim7	dim8
evec1	**0.998^a^**	0.01	-0.02	0.01	0.005	0.01	-0.002	-0.0004
evec2	0.00	**0.98**	0.17	0.03	-0.01	-0.01	0.01	0.002
evec3	-0.04	0.17	-0.96	-0.11	-0.01	0.01	0.01	-0.01
evec4	-0.02	0.00	-0.12	**0.89**	0.06	0.17	-0.07	-0.03
evec5	0.02	-0.01	-0.05	0.38	-0.20	-0.43	0.20	0.03
evec6	0.01	0.01	0.02	0.02	0.13	0.00	0.08	-0.17
evec7	-0.02	-0.001	-0.03	-0.01	0.17	-0.07	-0.17	0.31
evec8	0.02	-0.01	0.02	0.02	-0.26	-0.18	-0.32	0.06

^a^Bold font indicates the absolute value of correlation coefficient > 0.8.

### GWAS results

**Figure 2:**
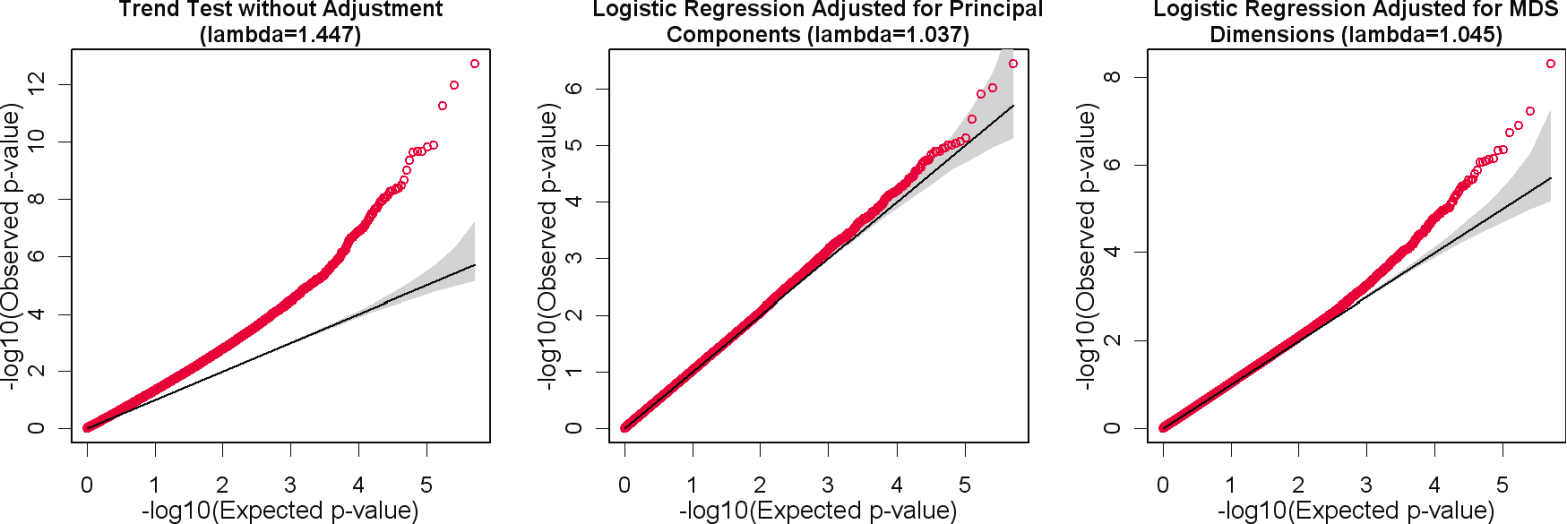
**Q-Q plots of *p*-values from three GWA analyses**. SNPs in HLA region are excluded to enhance readability.

The quantile-quantile (Q-Q) plots of the *p*-values from the three GWA analyses, as well as the corresponding genomic inflation factors of the three analyses, are presented in Figure 2. SNPs in the HLA region are excluded from the plots to enhance readability. Both the genomic inflation factor (λ) and the Q-Q plot of the analysis using trend test indicated a strong PS effect on the association result. The genomic inflation was 1.447, and the Q-Q plot deviated from the expected line from the beginning. This PS effect was successfully corrected by using logistic regression with the significant principal components as covariates. The genomic inflation factor fell to 1.037, and the *p*-values of majority of the SNPs fell between or very close to their 95% "concentration bands" (gray shaded area). Although the analysis adjusted for the leading MDS dimensions was able to reduce the genomic inflation factor to 1.045, the Q-Q plot of this analysis still showed an obvious deviation from the expected line, indicating an uncorrected *PS *effect.

**Figure 3:**
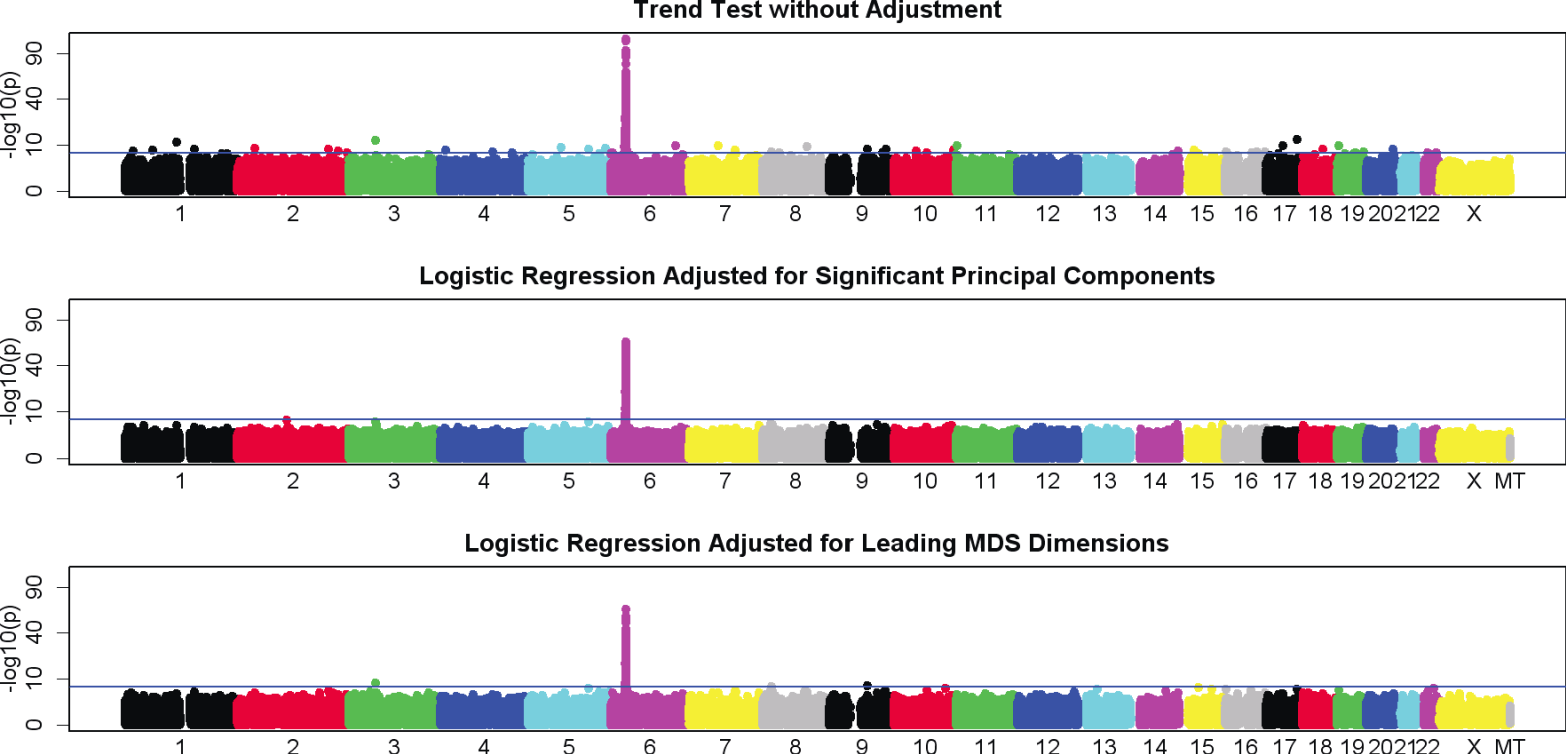
**Results of GWA analyses**. The y axis is in square root scale to enhance readability.

Results from the three GWA analyses were also plotted against their chromosome locations (Figure 3). All three analyses were able to identify the HLA region on chromosome 6, which had been implicated by numerous RA studies [8-11]. The total number of SNPs that reached genome-wide significance (*p*-value < 0.05/515,741) was 381 for the analysis using the trend test, 221 for the analysis adjusted for the significant principal components, and 194 for the analysis adjusted for the leading MDS dimensions. The majority of these significant SNPs were from the HLA region. SNP rs2476601 in the *PTPN22 *gene is a non-HLA SNP that had been associated with risk of RA [12]. Table 2 summarizes the *p*-values of this SNP from the three analyses as well as their corresponding rankings in non-HLA SNPs. After PS correction using either significant principal components or leading MDS dimensions, the *p*-value of this SNP became less significant and the ranking dropped, suggesting that PS may have also contributed to the previously reported association signals at this SNP.

**Table 2:** *p*-Values at SNP rs2476601 from three GWA analyses and their rankings in non-HLA SNPs

Analysis method	*p*-Value	Rank in non-HLA SNPs
Trend test without adjustment	5.42 × 10^-12^	3
Logistic regression adjusted for principal components	0.000018	18
Logistic regression adjusted for MDS dimensions	0.000022	59

Although PCA performed slightly better than MDS in correcting PS in the GWA analysis of this dataset, it would be inappropriate to conclude that PCA is a preferred approach in all GWAS. MDS is a more flexible method in general as compared with PCA. First, PCA requires that underlying data follow a multivariate normal distribution, while MDS imposes no such restriction. Second, PCA requires computation of a covariance matrix first, while MDS can be applied to any kind of distances or similarities. Pairwise IBS distance is only one example of many distance measures to which MDS can be applied. As a special case, MDS can be applied to the covariance matrix used in PCA as well. In this case, the performance of MDS in correcting PS will be equivalent to it of PCA [4].

## Conclusion

In this paper, we compared the performance of PCA and MDS in identifying population structure and correcting for PS in GWAS using data provided to GAW16 participants by the NARAC. While the two methods identified similar population structures in this dataset, PCA performed slightly better than MDS in correcting for PS in the GWA analyses of this data set.

## List of abbreviations used

GAW16: Genetic Analysis Workshop 16; GWA: Genome-wide association; GWAS: GWA studies; IBS: Identity-by-state; LD: Linkage disequilibrium; MDS: Multidimensional scaling; NARAC: North American Rheumatoid Arthritis Consortium; PCA: Principal-component analysis; PS: Population stratification; Q-Q: Quantile-quantile; RA: Rheumatoid arthritis; SNP: Single-nucleotide polymorphism.

## Competing interests

The authors declare that they have no competing interests.

## Authors' contributions

DW participated in discussions of the analytical approach, carried out the majority of the data analyses, and drafted the manuscript. YS participated in discussions of the analytical approach and carried out some of the analyses related to multi-dimensional scaling. PS, JAB, and MAW contributed to acquisition of the data and editorial revision of the manuscript. QL participated in discussions of the analytical approach.
